# Photoelectron Properties and Organic Molecules Photodegradation Activity of Titania Nanotubes with Cu_x_O Nanoparticles Heat Treated in Air and Argon

**DOI:** 10.3390/molecules27228080

**Published:** 2022-11-21

**Authors:** Elizaveta Konstantinova, Timofey Savchuk, Olga Pinchuk, Ekaterina Kytina, Elizaveta Ivanova, Lidiya Volkova, Vladimir Zaitsev, Alexander Pavlikov, Elena Elizarova

**Affiliations:** 1 Physics Department, M.V. Lomonosov Moscow State University, Moscow 119991, Russia; 2 Institute of Nano-, Bio-, Information, Cognitive and Socio-Humanistic Sciences and Technologies, Moscow Institute of Physics and Technology (National Research University), Moscow 141701, Russia; 3 A.M. Prokhorov General Physics Institute of RAS, Moscow 119991, Russia; 4 Institute of Advanced Materials and Technologies, National Research University of Electronic Technology—MIET, Moscow 124498, Russia; 5 Institute of General and Ecological Chemistry, Lodz University of Technology, 90-924 Lodz, Poland; 6 Phystech School of Electronics, Photonics and Molecular Physics, Moscow Institute of Physics and Technology (National Research University), Moscow 141701, Russia; 7 Department of Structural Analysis and Metrology, Institute of Nanotechnology of Microelectronics of the Russian Academy of Sciences, Moscow 119991, Russia; 8 Department of Food Hygiene and Toxycology, Institute of Vocational Education, I.M. Sechenov First Moscow State Medical University (Sechenov University), Moscow 119435, Russia

**Keywords:** organic molecules photodegradation, TiO_2_ nanotubes/Cu_x_O, nanoheterostructures, photocatalytic activity, molecular oxygen anion radicals, copper ions

## Abstract

Titania is very famous photocatalyst for decomposition of organic pollutants. Its photocatalytic properties significantly depend on the morphology and chemical composition of the samples. Herein, the TiO_2_ nanotubes/Cu_x_O nanoheterostructures have been synthesized and the effect of heat treatment performed in molecular atmospheres of air and argon on their photoelectrochemical and photocatalytic properties has been studied. The prepared samples have a higher reaction rate constant compared to TiO_2_ nanotubes in the decomposition reaction of methylene blue molecules. It is established that in argon treated nanoheterostructures, the copper oxide is present in two phases, CuO and Cu_2_O, while in air treated ones there is only CuO. In the TiO_2_ nanotubes/Cu_x_O samples, Cu^2+^ ions and molecular O_2_^−^ radicals were detected while in TiO_2_ nanotubes only carbon dangling bond defects are present. The dynamics of O_2_^−^ radicals under illumination are discussed. It was shown that the TiO_2_ nanotubes do not exhibit photocatalytic activity under visible light. The mechanism of the photocatalytic reaction on the surface of the TiO_2_ nanotubes/Cu_x_O samples was proposed. It is assumed that a photocatalytic decomposition of organic molecules under visible light at the surface of the nanoheterostructures under investigation is realized mainly by the reaction of these molecules with photogenerated O_2_^−^ radicals. The results obtained are completely original and indicate the high promise of the prepared photocatalysts.

## 1. Introduction

Industrial development requires significant energy resources, which leads to environmental pollution with various toxic organic substances [1,2,3]. One of the solutions to this problem is the use of photocatalysis to decompose toxic organic pollutant molecules to carbon dioxide and water under the influence of sunlight [4].

TiO_2_ is widely studied and used as a photocatalyst for decomposition of organic molecules due to the successful location of energy levels, photocorrosion resistance, relative non-toxicity and low cost [1,2,3]. However, the band gap of TiO_2_ is 3.2 eV, which does not allow the wide use of this material in photocatalysis, due to the fact that the share of ultraviolet radiation is about 5% of the solar spectrum [5]. When using arrays of titanium anode oxide nanotubes, it is possible to control various geometric characteristics of this material, such as external and internal diameters, length, and wall thickness. The geometric characteristics of titanium dioxide nanotubes are determined by the parameters of the anodic process of formation. Moreover, the formation of TiO_2_ nanotubes (TiO_2_ NTs) array directly on titanium conductive substrate provides good electrical contact and more efficient transfer of charge carriers to the electrode compared to a powder system of nanoparticles [3].

It is known that doping of TiO_2_ NTs with transition metals and nonmetals can increase the response of TiO_2_ NTs in the visible wavelength range [6]. In addition, the photoactivity of TiO_2_ NTs in the visible wavelength range can be increased due to the formation of semiconductor heterostructures with narrow-band semiconductors [7]. The formed heterojunction leads to an increase in the lifetime of photogenerated charge carriers, due to their effective separation at the interface of two semiconductors [8,9].

Various transition metal oxides, in particular, copper oxides, are used to create TiO_2_/MeO_x_ heterojunctions. Copper oxides attract attention because of their band gap width, which is 1.2–2 eV [10]; the nanoheterojunction between TiO_2_ and copper oxide increases the photocatalytic activity in the visible radiation range. Copper oxides can be obtained in various ways: microwave irradiation [11,12], hydrothermal synthesis [13,14], solvothermal [15], chemical deposition in a bath [16,17] pyrolysis by spraying [18,19], ion layering (SILAR) [20,21,22,23,24,25], sol-gel [26,27]. Among them, the SILAR method attracts a lot of attention due to its relatively low cost and the absence of the need to use complex equipment.

However, due to the presence of two stable phases of copper oxide (Cu_2_O, CuO), it becomes difficult to control the properties of the TiO_2_/Cu_x_O heterojunction. Various heat treatments of the obtained samples are considered by different scientific groups, describing several stoichiometric compositions of copper oxide attained depending on the temperature of heat treatment. For example, in [28], such samples were obtained where Cu_2_O is formed at lower temperatures, followed by Cu_2_O/CuO with increase of the temperature, and then CuO; with each increase in temperature, the Cu_2_O phase gradually decreased. In [29], the effect of the number of SILAR deposition cycles on the resulting copper oxide structure was investigated. In that study, no heat treatment was performed, but the sample was left at ambient temperature. From the data obtained in the work, it follows that with this manufacturing method, CuO oxide is deposited on the surface of TiO_2_. The authors of [30] describe the study of the decomposition of tartrazine dyes by combining the processes of electrocoagulation and photocatalysis. To evaluate these processes, a sample with TiO_2_ nanotubes and copper oxide particles deposited on them by the SILAR method was used. In the present work, heat treatment was carried out at a temperature of 500 °C for 3 h in air to obtain a stable crystalline structure of copper oxide. At this temperature CuO was also formed. In [31], a similar heat treatment at 450 °C for 3 h in air was used to crystallize copper oxide. At this time, Cu_x_O nanoparticles were inside TiO_2_ nanotubes. The results obtained show that the presence of CuO nanoparticles causes a decrease in the optical band gap of TiO_2_ from 3.2 eV to 2.8 eV. In all the described works, annealing in air is used to control the copper oxide phase. At the same time, heat treatment in oxygen-free atmospheres after deposition of copper oxide nanoparticles by the SILAR method can contribute to the formation of TiO_2_/Cu_2_O or more complex TiO_2_/CuO/Cu_2_O heterojunctions. However, at present, only a small number of works are devoted to the study of the influence of heat treatment conditions on the performance of TiO_2_ NTS/Cu_x_O photocatalysts obtained by the SILAR method.

Therefore, the purpose of this work is to study the photocatalytic and photoelectron properties of synthesized TiO_2_/Cu_x_O nanoheterostructures depending on the conditions of heat treatment. Since defects (radicals) play an important role in photoelectron processes, investigation of the type and properties of defects in the obtained samples were carried out in parallel.

## 2. Results

### 2.1. Morphology

**Figure 1:**
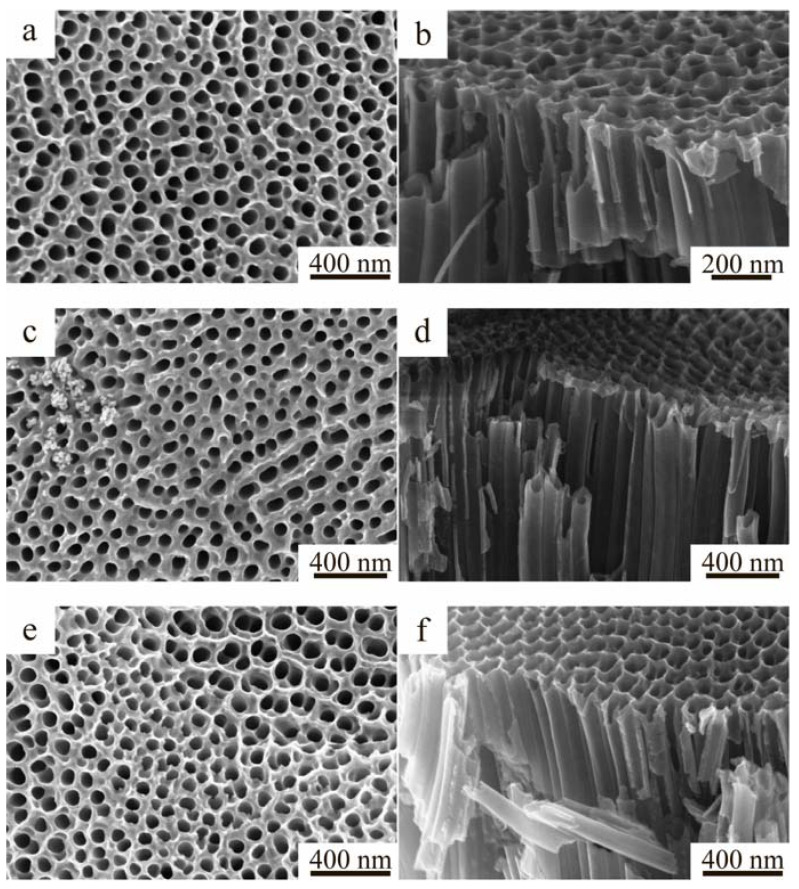
SEM images of the surface and chips of the obtained samples: (**a**,**b**) TiO_2_ NTs, (**c**,**d**) TiO_2_ NTs-S-Air, (**e**,**f**) TiO_2_ NTs-S-Arg.

The morphology of the formed TiO_2_ NTs and TiO_2_ NTs/CuO samples was studied using SEM. Figure 1 shows the photos obtained.

As can be seen from the microscopy results, there is an uneven distribution of copper oxide nanoparticles on the surface of titania nanotubes.

### 2.2. Structural Properties

To determine the phase of the copper oxides on the surface of TiO_2_ NTs, the samples obtained were examined by Time-of-Flight Secondary Ion Mass Spectrometry (TOF-SIMS). Well-separated peaks of ions of isotopes 63Cu^−^, 63CuO^−^, 63CuO_2_^−^ were observed. It was found that the ratio of 63CuO^−^, 63CuO_2_^−^ ions to the total number of registered ions is the largest for the TiO_2_-S-Air sample and exceeds that for the TiO_2_-S-Arg sample by 6 times. Based on the obtained results of the difference in the ratio of ion yield from the obtained samples, it can be concluded that the copper oxide on the surface of the TiO_2_-S-Air sample is in the more oxidized state compared to samples thermally treated in atmospheres with a low oxygen content. It can be assumed that the copper oxide phase in the TiO_2_-S-Air sample is more represented as CuO, whereas for TiO_2_-S-Arg samples, copper oxide located on the surface of TiO_2_ NTs can be represented as Cu_2_O or metallic copper.

### 2.3. Optical Properties

**Figure 2:**
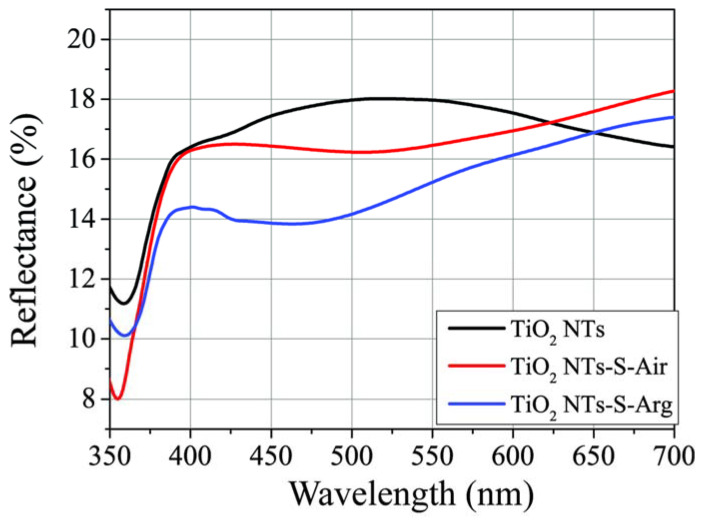
Diffuse light reflection spectra for different types of samples.

The diffuse light reflection method was used to study the optical properties (Figure 2). As can be seen from the figure, the initial pure TiO_2_ NTs mainly absorb UV light with a wavelength of less than 370 nm in accordance with the band gap width.

**Figure 3:**
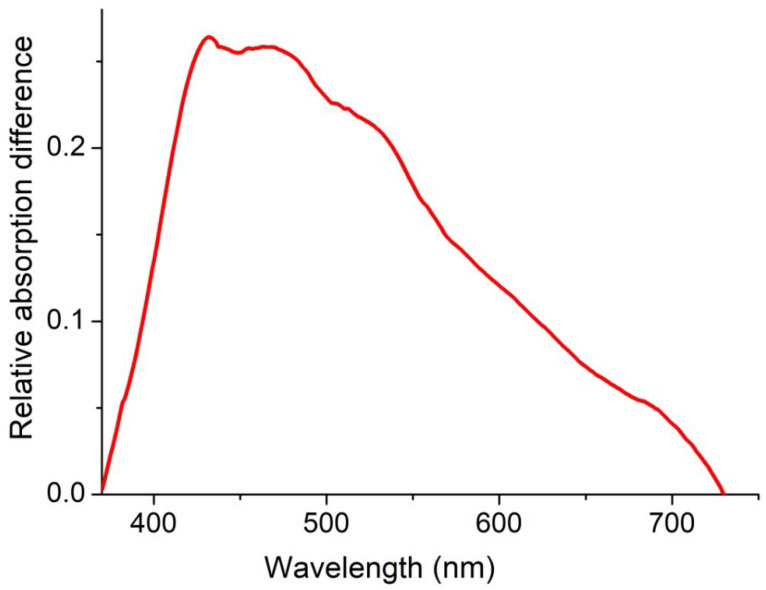
Relative excess absorption of TiO_2_-S-Arg samples compared to TiO_2_-S-Air samples.

After application of copper oxide nanoparticles, the light reflection coefficient from TiO_2_-S-Air and TiO_2_-S-Arg samples decreases in the visible range. Consequently, these samples absorb visible light better than the original ones. Compared with annealing in air, annealing in argon increases the absorption of visible light more significantly (Figure 3), which may be due to an increase in the concentration of defects.

**Figure 4:**
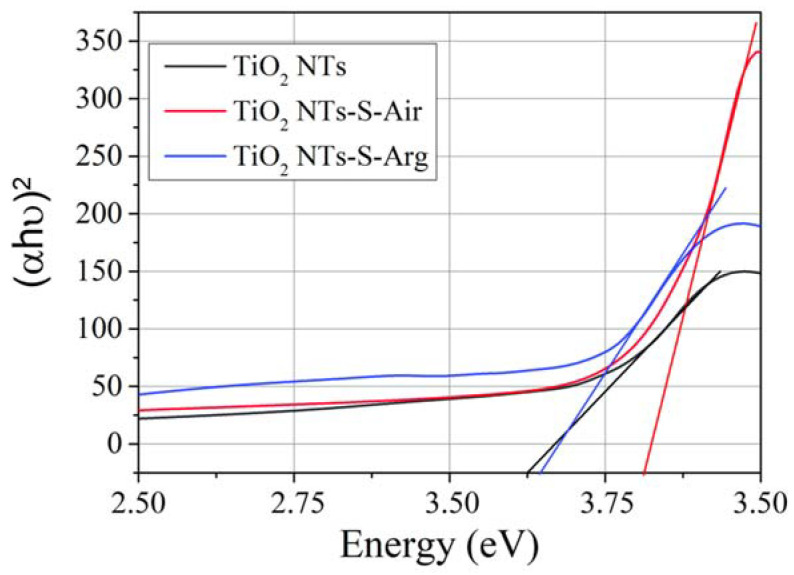
Determination of the forbidden band width for different samples according to the Kubelka–Munch theory.

The optical band gap of TiO_2_ NTs and TiO_2_ NTs/Cu_x_O was calculated using the Kubelka–Munch theory [32]. The corresponding graphical constructions are shown in Figure 4. The equations were used for semiconductors with direct band-to-band transitions. It was demonstrated earlier [33] that this approach provides reliable results for titanium-dioxide nanostructures. It is due to the size effect when crystal structure undergoes changes because of substance volume decrease; this results in an increased probability of direct band-to-band transitions. Conversion of an indirect semiconductor into a direct one is commonly described from the viewpoint of DFT theory [33].

The optical band gap of the initial TiO_2_ NTs annealed in air is about 3.2 ± 0.1 eV. After the introduction of copper oxide, the band gap practically did not change and was 3.3 ± 0.1 eV and 3.2 ± 0.1 eV, respectively, for samples annealed in air and in argon.

### 2.4. Photoelectrochemical Properties

**Figure 5:**
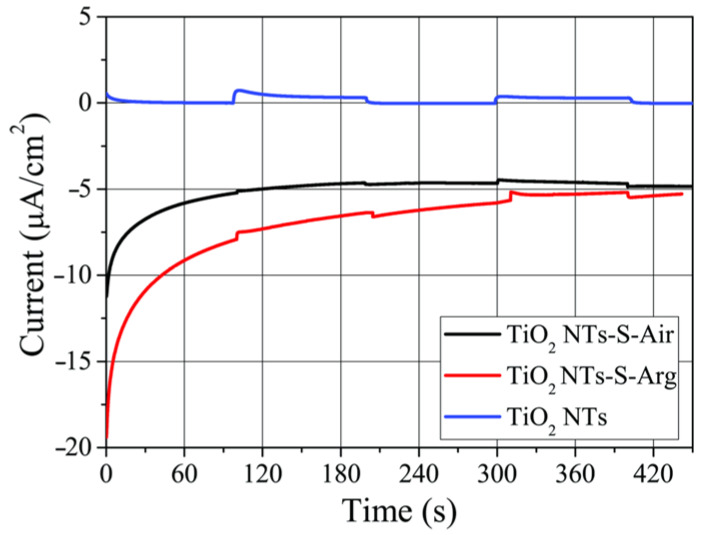
Photocurrent density kinetics of the samples annealed in different media and illuminated by visible light with a wavelength of more than 430 nm.

**Figure 6:**
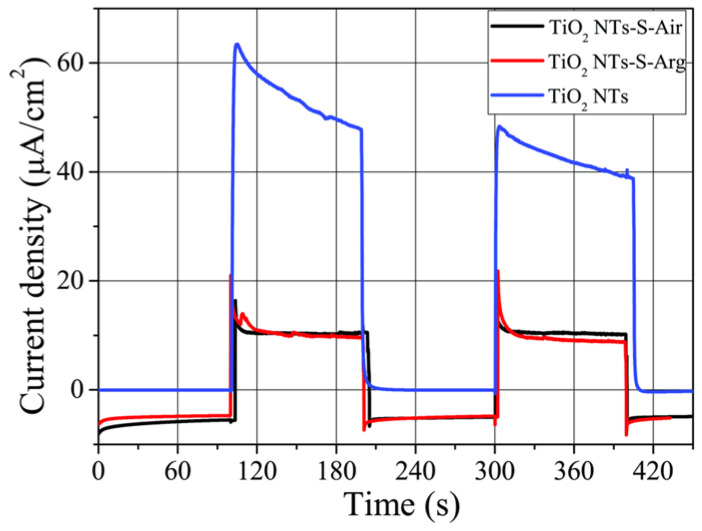
Photocurrent density kinetics for samples annealed in different media and under illumination in the wide light range using AM1.5 filter, imitating sunlight.

The photoactivity of the obtained samples was evaluated by chronoamperometry in a three-electrode photoelectrochemical cell (Figure 5 and Figure 6).

It can be seen from Figure 5 that the application of copper oxide does not lead to an increase in the generated photocurrent by TiO_2_ NTs arrays in the visible wavelength range. The photocurrent recorded in this region of the spectrum for all samples remains within the range of 1 μA/cm^2^. It is important to note that the dark current in 0.1 M solution of Na_2_SO_4_ for TiO_2_ NTs-S-Air and TiO_2_ NTs-S-Arg samples lies in the cathode region, which indicates the passage of a reduction reaction on the surface of the photocathode.

From Figure 6, it can be seen that the application of copper oxide leads to a decrease in the photoactivity of the samples under illumination over a wide range including UV light. The photocurrent values are about ~16 µA/cm^2^ and are almost the same for TiO_2_-S-Arg and TiO_2_-S-Air samples. The greatest value of the photocurrent 60 µA/cm^2^ is observed for the pure sample, TiO_2_ NTs, since titanium dioxide effectively absorbs UV light to generate free charge carriers. It is important to note that at the moment of switching on the light with the AM1.5 filter, no cathode current is observed on the samples decorated with copper oxide. This may be due to its insignificant value compared to the total anode current from TiO_2_ NTs samples.

### 2.5. Photocatalytic Activity and EPR Spectroscopy

**Figure 7:**
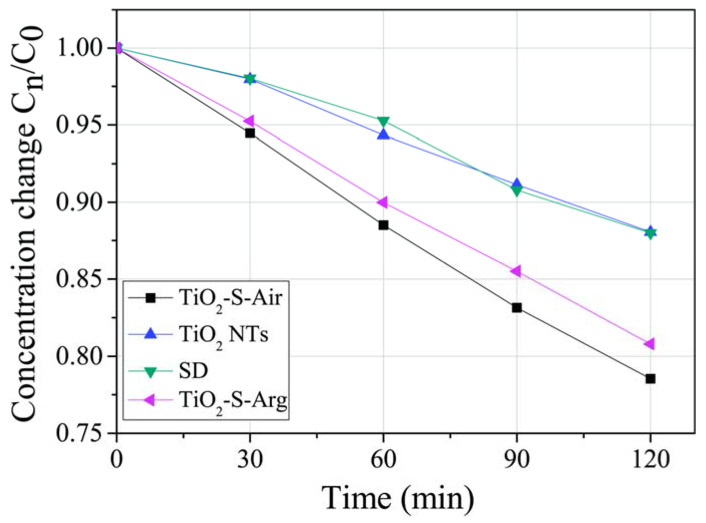
Kinetics of decomposition of methylene blue dye molecules on the surface of TiO_2_ NTs, TiO_2_-S-Air, TiO_2_-S-Arg samples and, for comparison, without a catalyst (SD) under illumination in the visible region.

To assess the photocatalytic activity with respect to organic molecules, the kinetics of decomposition of the methylene blue dye on the surface of TiO_2_ NTs/CuO samples were obtained. The degree of decomposition of the dye was estimated by the light transmission spectra. The obtained transmission data were recalculated into the relative concentration of the dye molecules C_n_/C_0_ (Figure 7), where C_0_ is the initial concentration.

From the data obtained, it can be concluded that the TiO_2_-S-Air and TiO_2_-SArg samples have the best photocatalytic properties (a change in the dye concentration of approximately 20%) comparing to the TiO_2_ NTs. The use of the TiO_2_ NTs sample did not add an effect compared to the self-degradation of methylene blue molecules under illumination (approximately 10%). This indicates the absence of catalytic activity of TiO_2_ NTs under visible light irradiation, which is consistent with the literature data.

**Figure 8:**
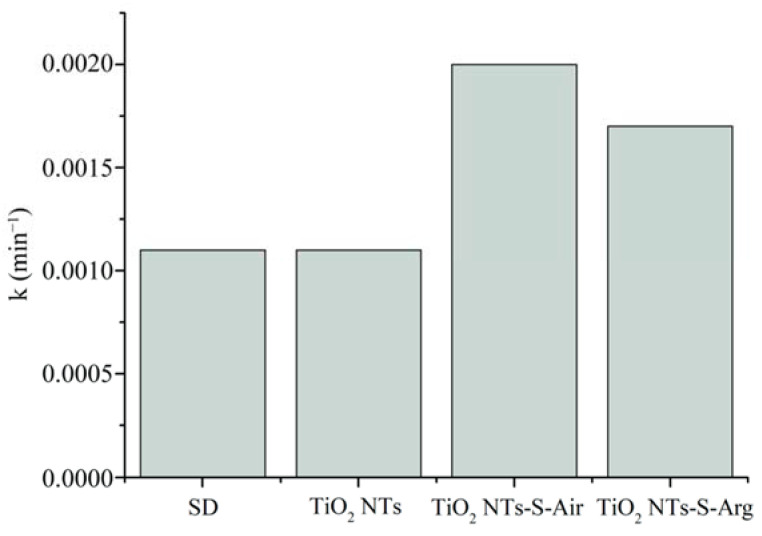
Rate constants of the decomposition reaction of methylene blue molecules using the obtained catalysts and without a catalyst (SD).

To evaluate the efficiency of methylene blue molecules decomposition by all the studied samples, the reaction rate constants were calculated. The TiO_2_-S-Air and TiO_2_-S-Arg samples have a high reaction rate constant (approximately 0.002). The smallest reaction rate constants, equal to 0.001, were found during dye self-degradation and when using TiO_2_ NTs (Figure 8).

**Figure 9:**
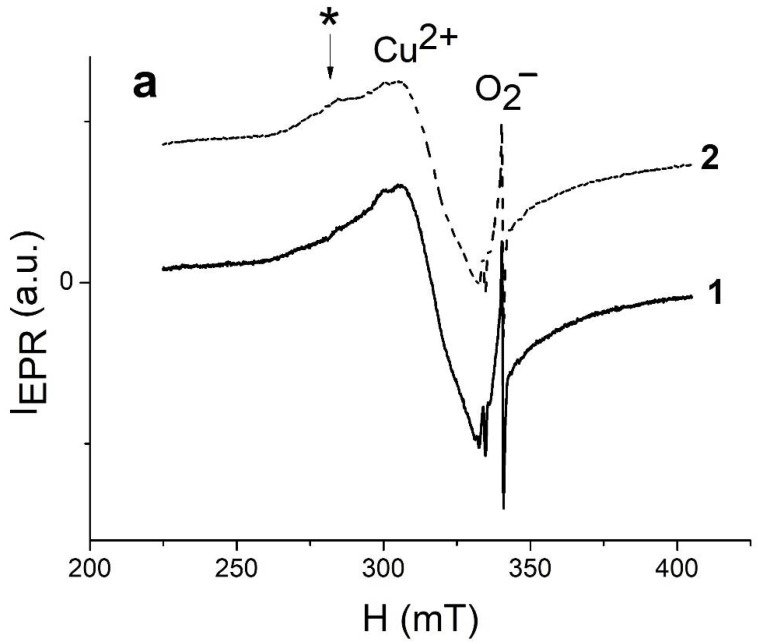

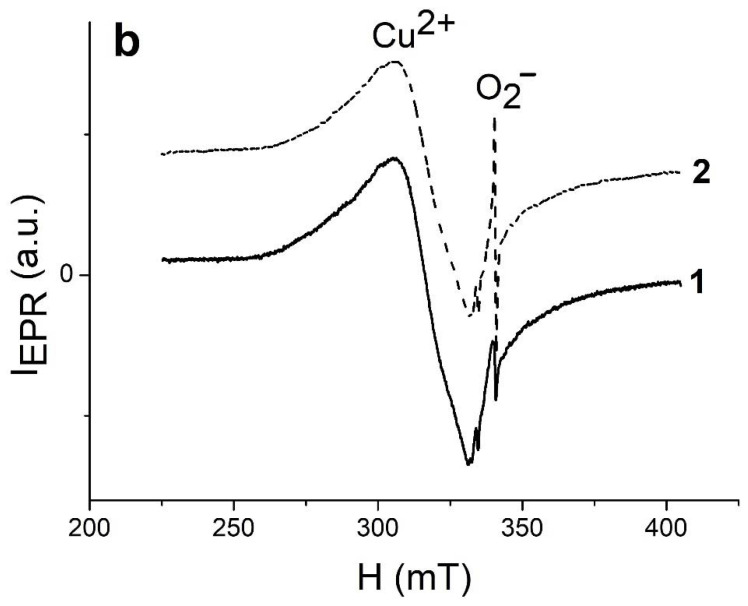
EPR spectra of (**a**) TiO_2_-S-Arg and (**b**) TiO_2_-S-Air samples in the dark (1) and under illumination (2). A “shoulder” of EPR signal marked with a symbol *, corresponds to the Cu^2+^ ions embedded in the TiO_2_ lattice during synthesis.

Before proceeding to the obtained results analysis and the identification of the mechanism of the photocatalytic process, it is necessary to study the structure of defects in the samples under study. Since most defects in metal oxides are paramagnetic, we used the electron paramagnetic resonance (EPR) method. The EPR spectra of TiO_2_-S-Air and TiO_2_-S-Arg nanocomposites are a superposition of several EPR signals (Figure 9).

First, a powerful EPR signal from Cu^2+^ copper ions (g = 2.1612) is recorded [34], which indicates the presence of the CuO phase. Besides, a signal from O_2_^−^ radicals is observed in the right part of the EPR spectra (Figure 9), (g_1_ = 2.029, g_2_ = 2.009, g_3_ = 2.003) [35]. The appearance of O_2_^−^ radicals can be easily explained by the adsorption of oxygen on the surface of the samples, followed by the capture of electrons from the conduction band. The intensity of the EPR signal from Cu^2+^ ions in TiO_2_-S-Arg samples is lower compared to TiO_2_-S-Air. This result is in good agreement with the data on TOF-SIMS, according to which, in TiO_2_-S-Arg samples, along with CuO, there is a Cu_2_O phase containing non-paramagnetic Cu^+^ ions. Thus, the smaller number of Cu^2+^ ions in TiO_2_-S-Arg can be explained by the presence of copper oxide in these structures in two phases: CuO and Cu_2_O. Under illumination, there is a decrease in the intensity of the EPR signal from Cu^2+^ ions in both TiO_2_-S-Air and TiO_2_-S-Arg samples, which can be explained by the transition of Cu^2+^ to Cu^+^, that is, the formation of the Cu_2_O phase [36]. The intensity of the EPR signal from molecular O_2_^−^ radicals before illumination is significantly higher in TiO_2_-S-Arg. This can be explained by the fact that the annealing of samples in an inert atmosphere is accompanied by the formation of oxygen vacancies on which oxygen molecules can be adsorbed, followed by capturing of electrons from the conduction band and the formation of molecular O_2_^−^ radicals. The phase change from CuO to Cu_2_O is accompanied by the formation of O_2_ molecules [37]; this oxygen can also be restored and contribute to the EPR signal from molecular O_2_^−^ radicals. Under the influence of illumination, photoinduced oxygen adsorption is initiated with the formation of molecular O_2_^−^ radicals; therefore, a sharp increase in the intensity of the EPR signal from molecular O_2_^−^ radicals in TiO_2_−S−Air is observed. However, there is no significant increase in the concentration of oxygen anion radicals in TiO_2_-S-Arg under illumination, which can be explained by the predominant generation of nonparamagnetic O_2_^2−^ ions in these samples due to the continuous capture of electrons: O_2_^−^ + e^−^ → O_2_^2−^. Such oxygen species are chemically less active than oxygen anion radicals and the photocatalysis rate of the TiO_2_-S-Arg samples does not exceed the photocatalysis rate of the TiO_2_-S-Air ones. We suppose that a transition from Cu_2_O to Cu is difficult at the surface of the TiO_2_-S-Arg, and no new oxygen molecules are released. This fact may also indicate that Cu_2_O is located predominantly on the surface. In the range of the magnetic field from 260 to 300 mT, a “shoulder” (marked with a symbol * in Figure 9a) on the EPR spectrum is observed, which is most pronounced for TiO_2_-S-Arg. According to data in the literature, EPR from Cu^2+^ copper ions embedded in the TiO_2_ lattice during synthesis is observed in this spectral range [36]. Notice that in TiO_2_ NTs without copper oxide nanoparticles only carbon dangling bond defects are detected [38]. Such defects in TiO_2_ nanotubes create energy levels in the band gap and are thus responsible for absorption in the visible range of the spectrum.

## 3. Discussion

**Figure 10:**
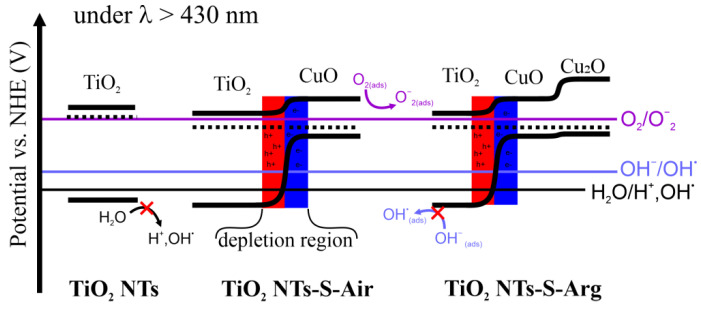
Energy band bending model for TiO_2_ NTs/Cu_x_O composites.

Based on the obtained results of the study of the photocurrent, photocatalytic activity, and the behavior of paramagnetic centers under illumination, the following model of the photocatalytic reaction mechanism on the surface of the obtained TiO_2_ NTs samples can be assumed (Figure 10). The relative position, the values of the energy band edges and reaction potentials are indicated in accordance with the literature data [39,40,41,42,43,44].

As a result of the spatial charge region formation at the TiO_2_/CuO interface, the current of the main charge carriers (e^−^) in n-type semiconductors is strongly suppressed, which is confirmed by the results of measurement of photocurrent dependencies. The region of spatial charge depleted by electrons (e^−^) in TiO_2_ can be comparable with the small (~50 nm) thickness of the walls of nanotubes, which can lead to almost complete blocking of the current through the volume of the nanotube. In the case when the depletion region formed at the TiO_2_/electrolyte interface completely overlaps TiO_2_, this does not prevent the reaction of photogenerated holes with adsorbed water molecules and OH^−^ ions. The photogenerated electrons move towards the cathode through an electrical circuit. In turn, the H^+^ ions formed during the water decomposition reaction migrate through the electrolyte volume and are reduced at the cathode, thereby closing the electrochemical circuit. In the case of the arrays of TiO_2_ nanotubes with CuO or Cu_2_O on their surface, the current of photogenerated holes will be directed to copper oxide. The valence band potential of copper oxide is insufficient for the water decomposition reaction to proceed: without additional bias the holes can only recombine in the volume of copper oxide without contributing to the photocurrent. However, this does not affect photocatalysis, because the mechanism of decomposition of organic substances in our case is different. Let us discuss it.

The process of organic molecules decomposition on the surface of semiconductors is often associated with the indirect decomposition of complex molecules due to the formation of highly active radicals O_2_^−^ and ${\mathrm{OH}}^{•}$ [45,46]. The formation of such radicals occurs due to the reaction of molecules of adsorbed oxygen, hydroxide ions OH^−^ and water molecules with photoinduced charge carriers, according to Reactions 1–3:

$${\mathrm{OH}}^{-}+{\;h}^{+}\to {\mathrm{OH}}^{•}$$

(1) $$E_{{\mathrm{OH}}^{-}/{\mathrm{OH}}^{•}}=1.47\;V\;\mathrm{vs}.\;\mathrm{NHE}\;\left(\mathrm{pH}=7\right)\;$$

$$H_{2}O+h^{+}\to H^{+}+{\mathrm{OH}}^{•}$$

(2) $$E_{H_{2}O/H^{+},{\mathrm{OH}}^{•}}=2.31\;V\;\mathrm{vs}.\;\mathrm{NHE}\;\left(\mathrm{pH}=7\right)$$

$$O_{2}+e^{-}\to O_{2}^{-}$$

(3) $$E_{O_{2}/O_{2}^{-}}=-0.18\;V\;\mathrm{vs}.\;\mathrm{NHE}\;\left(\mathrm{pH}=7\right)$$

Due to the fact that the study of photocatalytic activity was carried out with illumination by light with the wavelength longer than 430 nm, the maximum energy of the incident photon is ~2.9 eV. This energy is insufficient to generate electron-hole pairs in the TiO_2_ NTs sample (Eg ~ 3.2 eV), and therefore active reactions involving free charge carriers and adsorbed ions are impossible. In turn, to generate free charge carriers in CuO and Cu_2_O, the energy of the photon equal to 2.9 eV is sufficient.

The course of Reactions 1 and 2 on the surface of copper oxide nanoparticles is suppressed due to the more negative potential of the valence band top of both CuO and Cu_2_O and the potentials of Reactions 1 and 2. Therefore, the presence of free charge carriers in the near-surface layer of copper oxide nanoparticles cannot contribute to the course of these reactions.

In turn, the oxygen reduction Reaction (3) is thermodynamically possible on both semiconductors in the presence of free electrons. As shown by the EPR method, in the samples TiO_2_-S-Air and TiO_2_-S-Arg even without lighting, oxygen radicals O_2_^−^ necessary for the decomposition reaction of methylene blue molecules are present. When the light is turned on, the concentration of O_2_^−^ radicals for the TiO_2_-S-Air sample increases; as a result of a change in the CuO phase to Cu_2_O, O_2_ molecules can be released with its further reduction on the surface of copper oxide (I) to O_2_^−^. It can be assumed that due to the additional source of molecular O_2_^−^ radicals, the reaction rate constant of the TiO_2_-S-Air sample is slightly higher compared to TiO_2_-S-Arg. In turn, it is clearly shown that the TiO_2_ NTs sample without copper oxide and oxygen radicals does not exhibit photocatalytic activity, which is associated with the generation of O_2_^−^ on the surface of copper oxides. The results obtained indicate that the mechanism of photocatalytic decomposition of organic molecules under visible light illumination using TiO_2_/CuO-Cu_2_O nanoheterostructures is associated with the generation of O_2_^−^ on the surface of the samples and organic molecules’ reaction with O_2_^−^ radicals.

## 4. Materials and Methods

### 4.1. Synthesis of TiO_2_ NTs

Samples of titanium nanotube oxide (TiO_2_ NTs) were obtained by electrochemical oxidation of titanium. Electrolyte used: ethylene glycol, 0.3 g NH_4_F, 2 mL H_2_O per 100 mL volume of electrolyte. Anodizing was carried out in a thermostatically controlled cell at 20 °C in 2 stages. The first stage lasted 30 min, after which the formed nanotube layer was removed from the foil surface by cathodic polarization in a 5% H_2_SO_4_ solution. The second stage lasted 1 h, after which the sample was washed in ethyl alcohol and dried in an air stream.

After that, the obtained samples were subjected to heat treatment in a muffle furnace at 450 °C for 1 h for crystallization.

### 4.2. Synthesis of TiO_2_ NTs/Cu_x_O Heterostructures

CuO particles on the surface of TiO_2_ NTs were obtained by molecular layering (SILAR). The source of copper ions was an aqueous solution of CuCl_2_·2H_2_O, the pH of which was brought to 10 with a solution of 25% ammonia (NH_4_OH). As a source of anions, heated to 70 °C solution of ethyl alcohol with deionized water in a ratio of 1 to 3 was used.

The SILAR method consists of three stages. At the first stage, the sample was immersed for 30 s in an aqueous solution of copper chloride containing [Cu(NH_3_)_4_]^+2^ ions. At the second stage, the sample was placed in a solution of ethyl alcohol with deionized water for 7 s. The third stage consists of washing the sample in deionized water for 30 s. After the deposition process was completed, the obtained samples were subjected to heat treatment in a furnace at a temperature of 300 °C for 1 h to crystallize the deposited CuO layer in various annealing media (air, argon). We chose two different media for annealing—oxygen-rich and oxygen-free, in order to determine in which media the largest number of oxygen radicals (involved in photocatalytic oxidation reactions of organic substances) are formed on the sample surface.

Depending on the conditions of heat treatment, the designations of the samples in the work will be used as follows: TiO_2_ NTs—without copper oxide applied, TiO_2_-S-Air—annealing in air after applying copper oxide, TiO_2_-S-Arg—annealing in argon after applying copper oxide.

### 4.3. Microscopy

The surface morphology was studied using a Helios G4CX (Thermo Fisher Scientific, Waltham, MA, USA) scanning electron microscope.

### 4.4. Time-of-Flight Secondary Ion Mass Spectrometry

The Time-of-Flight Secondary Ion Mass Spectrometer was produced by TOF-SIMS IV manufactured by IONTOF GmbH, Muenster, Germany, and clusters of bismuth ion (Bi^3+^) were used.

### 4.5. Investigation of Optical Properties

The obtained samples were examined by diffuse light reflection in the wavelength range from 200 to 800 nm using the spectrometer (LS-55 PerkinElmer, St Waltham, MA, USA). The values of the optical band gap width were obtained using the mathematical transformation based on Kubelka–Munch theory.

### 4.6. Photoelectrochemical Properties

To assess the photoelectrochemical properties, the Zolix SCS10-PEC-Pro (Tongzhou District, Beijing, China) photoelectrochemical research unit was used, which consisted of a 150 mL photoelectrochemical cell equipped with an Ag/AgCl (3M) reference electrode and a platinum counterelectrode. Chronoamperograms were obtained at 0 V bias vs. Ag/AgCl (3M). A 500 W xenon lamp was used as a light source. The photoactivity of the samples was studied in the visible range λ > 430 nm, for which a UV filter (Photooptic-filters, Obninsk, Russia) was used, and AM1.5 filter (Zolix Instruments, Beijing, China) was also used to study the samples in a spectrum similar to the solar one. The incident light power was about 100 mW/cm^2^ and 80 mW/cm^2^, respectively. An aqueous solution of 0.1 M Na_2_SO_4_ was used as the electrolyte.

### 4.7. Photocatalysis

A Newport xenon Lamp 150W Xe (Deere Avenue Irvine, CA, USA) with a UV filter was used as a light source (to highlight the visible range: λ > 430 nm), the incident light power was ~100 mW/cm^2^.

For the study of photocatalytic treatment of organic molecules, methylene blue (Methylenum coeruleum) was used in an aqueous solution of 6.7 μM concentration and a volume of 20 mL. To avoid severe degradation of methylene blue molecules due to heating by lamp radiation, an optimal temperature of about 20 °C was maintained using a circulating thermostat.

Light transmission through the aqueous solution of methylene blue was measured using a spectrophotometer SF-102 (“NPO Akvilon”, Podolsk, Russia) at 662 nm wavelength, since this corresponds to the peak absorption of the studied solution. Before the photocatalysis process, the sample was placed in 20 mL of the prepared solution of methylene blue for 1 h for preliminary adsorption of the dye onto the sample surface. Light transmission through the solution before soaking the sample was also measured, which in all cases was about 30%. After that, the light transmission through the solution was measured after soaking the sample (zero point). Then the photocatalysis process was started and the light transmission through the decomposed solution was measured on the spectrophotometer every 30 min for 2 h.

### 4.8. EPR Spectroscopy

Electron paramagnetic resonance (EPR) spectra were recorded on a Bruker ELEXSYS E500 EPR spectrometer (X-band) (Bruker, Karlsrue, Germany). The samples were illuminated directly in the cavity of the EPR spectrometer with the light of a BRUKER ELEXSYS ER 202 UV high-pressure mercury lamp (50 W). The photoexcitation intensity of the samples was approximately 100 mW/cm^2^.

## 5. Conclusions

The TiO_2_ nanotubes/Cu_x_O nanoheterostructures formed with heat treatment in molecular atmospheres of air and argon have been prepared and investigated using microscopy, TOF-SIMS, UV-Vis spectroscopy, chronoamperometry, photocatalysis and EPR spectroscopy. The TiO_2_ nanotubes have been synthesized separately for comparative study. The TiO_2_ nanotubes/Cu_x_O nanoheterostructures were characterized by better photocatalytic properties regarding dye decomposition (a change in the dye concentration was of approximately 20%) compared to the titania nanotubes (a change in the dye concentration was on the level of self-degradation of methylene blue molecules under illumination). It was revealed that in air treated nanoheterostructures the copper oxide was present in the CuO phase, but in argon treated samples two phases, both CuO and Cu_2_O, were detected. In the TiO_2_ nanotubes/Cu_x_O samples, Cu^2+^ ions and molecular O_2_^−^ radicals were observed while in TiO_2_ nanotubes only carbon dangling bond defects were detected. The intensity of the EPR signal from Cu^2+^ ions in TiO_2_ nanotubes/Cu_x_O samples decreased under illumination; this can be explained by the transition of Cu^2+^ to Cu^+^ state, that is, the formation of the Cu_2_O phase. Note that, without illumination, the amount of oxygen radicals was higher in the samples that underwent heat treatment in argon, but under illumination, the amount of oxygen radicals became approximately the same for both types of samples. Therefore, there are two ways to achieve a high concentration of oxygen radicals necessary for the photodegradation activity in visible light of the TiO_2_ nanotubes/CuxO nanoheterostructures: (1) annealing in oxygen-enriched media followed by illumination or (2) simple annealing in an oxygen-free media. It was also shown that the TiO_2_ nanotubes without copper oxide and molecular oxygen radicals did not exhibit photocatalytic activity in the same conditions. The mechanism of photocatalytic decomposition of the test dye under visible light illumination using TiO_2_/CuO-Cu_2_O nanoheterostructures was discussed and associated with the generation of $O_{2}^{-}$ radicals on the surface of the samples and the involvement of $O_{2}^{-}$ radicals in redox reactions with dye molecules.

The results obtained are completely new and give us every reason to consider the prepared TiO_2_ nanotubes/Cu_x_O nanoheterostructures with high concentrations of oxygen radicals as promising photocatalysts.

## Data Availability

Not applicable.

## Sample Availability

Samples of TiO_2_ NTs, TiO_2_-S-Air, and TiO_2_-S-Arg are available from the authors.
